# The Effect of Highly Active Antiretroviral Therapy on the Survival of HIV-Infected Children in a Resource-Deprived Setting: A Cohort Study

**DOI:** 10.1371/journal.pmed.1001044

**Published:** 2011-06-14

**Authors:** Andrew Edmonds, Marcel Yotebieng, Jean Lusiama, Yori Matumona, Faustin Kitetele, Sonia Napravnik, Stephen R. Cole, Annelies Van Rie, Frieda Behets

**Affiliations:** 1 Department of Epidemiology, University of North Carolina at Chapel Hill, Chapel Hill, North Carolina, United States of America; 2 School of Public Health, University of Kinshasa, Kinshasa, Democratic Republic of the Congo; 3 School of Medicine, University of North Carolina at Chapel Hill, Chapel Hill, North Carolina, United States of America; Massachusetts General Hospital, United States of America

## Abstract

**Background:**

The effect of highly active antiretroviral therapy (HAART) on the survival of HIV-infected children has not been well quantified. Because most pediatric HIV occurs in low- and middle-income countries, our objective was to provide a first estimate of this effect among children living in a resource-deprived setting.

**Methods and Findings:**

Observational data from HAART-naïve children enrolled into an HIV care and treatment program in Kinshasa, Democratic Republic of the Congo, between December 2004 and May 2010 were analyzed. We used marginal structural models to estimate the effect of HAART on survival while accounting for time-dependent confounders affected by exposure. At the start of follow-up, the median age of the 790 children was 5.9 y, 528 (66.8%) had advanced or severe immunodeficiency, and 405 (51.3%) were in HIV clinical stage 3 or 4. The children were observed for a median of 31.2 mo and contributed a total of 2,089.8 person-years. Eighty children (10.1%) died, 619 (78.4%) initiated HAART, six (0.8%) transferred to a different care provider, and 76 (9.6%) were lost to follow-up. The mortality rate was 3.2 deaths per 100 person-years (95% confidence interval [CI] 2.4–4.2) during receipt of HAART and 6.0 deaths per 100 person-years (95% CI 4.1–8.6) during receipt of primary HIV care only. The mortality hazard ratio comparing HAART with no HAART from a marginal structural model was 0.25 (95% CI 0.06–0.95).

**Conclusions:**

HAART reduced the hazard of mortality in HIV-infected children in Kinshasa by 75%, an estimate that is similar in magnitude but with lower precision than the reported effect of HAART on survival among children in the United States.

*Please see later in the article for the Editors' Summary*

## Abstract 2

This observational cohort study by Andrew Edmonds and colleagues reports that treatment with highly active antiretroviral therapy (HAART) markedly improves the survival of HIV-infected children in Kinshasa, DRC, a resource-deprived setting.

## Editors' Summary

**Background:**

In 2009, an estimated 2.5 million children were living with HIV, the majority of whom (2.3 million) were in sub-Saharan Africa. Most (90%) of these children acquired HIV from their HIV-infected mothers during pregnancy, birth, or breastfeeding, highlighting the importance of giving effective drugs for the prevention of mother to child transmission. As such interventions are still not widely accessible or available in most resource-limited countries, where the burden of HIV is highest, every day an estimated 1,000 children were newly infected with HIV in 2009, but only 360,000 children were receiving highly active antiretroviral therapy (HAART).

Although HAART improves the survival of adults living with HIV, less is known about the degree to which HAART affects the survival of HIV-infected children—although response to antiretroviral treatment is known to differ across age groups. Furthermore, as the course of HIV disease in children is different from that in adults (partly because of the impact of the virus on the immature thymus, which can lead to high HIV RNA viremia and rapid death), it is inappropriate to extrapolate results from studies of adults to pediatric populations. Therefore, it is imperative that the effect of HAART on survival be quantified specifically in children.

**Why Was This Study Done?:**

Most observational studies of the effects of treatment on child survival have been undertaken in high-income countries, such as Italy and the United States. As most children with HIV live in low-resource areas, where multiple factors, such as delayed presentation to care and a higher incidence of co-occurring conditions, might adversely affect treatment outcomes, there is a specific need for information on the effects of HAART in children with HIV living in low-income countries. Although some investigations have taken place in pediatric cohorts from such countries (for example, Côte d'Ivoire, Haiti, Lesotho, Thailand, and Zambia), the effect of HAART on mortality has not been accurately quantified among children in a resource-deprived setting. Therefore, in this observational clinical cohort study, the researchers investigated the effect of HAART on mortality in HIV-infected children in Kinshasa, in the Democratic Republic of the Congo (DRC).

**What Did the Researchers Do and Find?:**

The researchers analyzed data from 790 children enrolled into an HIV program in Kinshasa, DRC, between December 2004 and May 2010 and used a statistical model (marginal structural models) to adjust for time-dependent confounding factors, such as the fact that HAART is typically initiated in sicker patients, for example, those with lower CD4 cell percentages. Assuming that all children starting HAART received it uninterruptedly throughout follow-up, using this statistical model, the researchers were able to compare the hazard ratio of death had all children initiated HAART to that had no children initiated HAART during follow-up.

In the study, 619 out of the 790 children (78.4%) initiated HAART during follow-up and were followed for a median of 31.2 months, with a median of 30 HIV care visits. Of those who started treatment, 110 (17.8%) switched to an alternative regimen because of an adverse event or treatment failure. During the 2,089.8 accrued person-years of follow-up, 80 children (10.1%) died, giving an overall mortality rate of 3.8 deaths per 100 person-years. The unadjusted mortality rate ratio comparing HAART to no HAART was 0.54. Using a marginal structural model, the researchers estimated that compared to no HAART, HAART reduced the hazard (rate) of mortality during follow-up by 75%.

**What Do These Findings Mean?:**

These findings show that treatment with HAART markedly improved the survival of children infected with HIV in Kinshasa, DRC, and suggest that HAART is as effective in improving the survival of HIV-infected children in a severely resource-deprived country (still recovering from civil war) as in more resource-privileged settings—an important finding given that the vast majority of children receiving HAART live in resource-poor areas. This study provides additional evidence that accelerating rollout of antiretroviral therapy to children with HIV in resource-poor countries is lifesaving and effective. Future research needs to address how effective HAART is in understudied populations in resource-poor countries, such as undernourished children or those with co-infections such as tuberculosis.

**Additional Information:**

Please access these Web sites via the online version of this summary at http://dx.doi.org/10.1371/journal.pmed.1001044.

- The World Health Organization's Web site has more information about the treatment of children living with HIV
- Médecins Sans Frontières's Campaign for Access to Essential Medicines Web site has more information on pediatric HAART

## Introduction

Highly active antiretroviral therapy (HAART) clearly improves the survival of adults living with HIV [1],[2], even when initiated at higher CD4 cell counts [3],[4], but less is known about the degree to which HAART affects the survival of HIV-infected children. The course of HIV disease in children, in part because of deleterious impacts of the virus on the immature thymus [5], leading to high HIV RNA viremia [6] and rapid death [7], is distinct from that in adults [8]. Response to antiretroviral treatment also differs across age groups [9]. Given that the natural history of HIV and response to therapy vary by age and that more than two million children worldwide are living with HIV [10], the extrapolation of results from studies of adults to pediatric populations is not appropriate. It is imperative that the effect of HAART on survival is specifically quantified in children.

Most observational studies on the effects of treatment on survival in children have not used the epidemiological methods necessary to account for potential biases inherent to their design, including confounding by indication. One study not employing methods necessary to account for such possible biases found that HAART, relative to no therapy, decreased the mortality rate by 71% among 1,142 Italian children [11]. A recent study of 1,236 children in the United States [12], which used optimal analytical methods, observed a result similar to that of the Italian study.

More than 90% of children receiving HAART live in low-resource areas [10],[13]. Because multiple factors that may adversely affect treatment outcomes including delayed presentation to care and a higher incidence of co-occurring infectious and non-infectious conditions such as undernutrition are more common in such environments [14], there is a specific need for information on the effects of HAART on patients living in these areas. Studies in adults have revealed higher mortality after HAART initiation in resource-poor settings than in resource-privileged settings after adjusting for age, CD4 cell count, and disease stage [15],[16]. Although investigations in pediatric cohorts from Zambia [17], Haiti [18], and Côte d'Ivoire [19] have shown that treatment improves immunological, hematological, and growth outcomes, and results in mortality rates lower than those observed in the pre-antiretroviral era [7], an estimate of the effect on mortality of HAART, compared to no HAART, has never been accurately quantified among children in a resource-deprived setting. This is true of two recent multi-site studies in sub-Saharan Africa [20],[21], as well as studies in Thailand [22], Zambia [23], Côte d'Ivoire [24], and Lesotho [25] that have provided valuable information on mortality among children receiving HAART, including rates during the early and late therapeutic periods.

In this study, we investigated the effect of HAART on mortality in an observational clinical cohort of HIV-infected children in the Democratic Republic of the Congo (DRC). As HAART is typically initiated in sicker patients, for example, those with lower CD4 cell counts, it is necessary to adjust for this confounding by indication to estimate its effect on survival. But adjusting for a time-dependent factor such as CD4 cell count that is itself affected by prior HAART exposure can yield a measure not interpretable as the total effect of HAART, because the effects of therapy are mediated in part through modulation of CD4 count. We therefore used a method that adjusts for time-dependent confounding by indication while accounting for confounders affected by prior exposure: marginal structural models.

## Methods

### Ethics Statement

Parental informed consent for the HIV care program, in addition to assent from minors 12 y of age or older, was obtained for all participants. All research was conducted according to the principles expressed in the Declaration of Helsinki and was approved by the Ethics Committee of the Kinshasa School of Public Health (approvals ESP/CE/010 and ESP/CE/014) and the University of North Carolina at Chapel Hill Institutional Review Board (studies 04-1007, 05-2311, and 10-0661).

### Study Population, Measurements, and Follow-up

The source of information for this study was a comprehensive HIV care and treatment program serving children and family members at Kalembe Lembe Pediatric Hospital and Bomoi Healthcare Center in Kinshasa, DRC [26]. HIV infection was confirmed by serological testing, with HIV viral load or DNA PCR used in infants under 18 mo of age. Patients were managed in accordance with World Health Organization (WHO) [27],[28] and national [29] guidelines regarding diagnosis and treatment of opportunistic infections, laboratory monitoring, and provision of cotrimoxazole and HAART when clinically or immunologically indicated. HAART could be initiated as soon as 1 wk following enrollment or at any visit thereafter, with the standard first-line regimen comprising zidovudine or stavudine, lamivudine, and nevirapine (NVP) or efavirenz. Prior to October 2009, zidovudine, lamivudine, and NVP were given as syrups and dosed according to milligrams per kilogram or body surface area, as appropriate, per package inserts. From October 2009 on, pediatric fixed-dose combination tablets were administered based on WHO-recommended weight-banded dosing. Because virological diagnostics were occasionally unavailable, enrolled infants under 2 y of age did not always begin treatment immediately as recommended by WHO [28]. Visits were scheduled monthly for patients receiving HAART and quarterly for those not receiving HAART, with additional unscheduled visits made by individuals needing acute care. Clinical data, documented by physicians during patient visits using standardized forms, were collected. CD4 cell count and percentage were evaluated every 6 mo at the DRC National AIDS Reference Laboratory, but viral load was not routinely assessed because of infrequent availability of the assay.

The population for this analysis was HIV-infected children who were naïve to antiretroviral therapy and under 18 y of age at the start of follow-up. The beginning of follow-up, also referred to as baseline, was the initiation of HIV care (between December 2004 and May 2010) unless a child's CD4 percentage was not obtained at that visit, in which case baseline was defined as the date of the first available CD4 percentage result. Consistent with precedent [12],[30], allowing follow-up to start at first CD4 percentage meant that children did not have to be excluded due to missing immunological data. Follow-up ended at death or at the last clinic visit prior to transfer to a different care provider, loss to follow-up, or 1 August 2010. If children withdrew from care or could not be located by three tracking attempts after a missed visit, they were classified as lost to follow-up. Children contributed non-HAART person-time until they initiated HAART. Program personnel gathered information on children who died, including the date and suspected cause of death.

### Statistical Analysis

Mortality rates were expressed as deaths per 100 person-years, with rates and rate ratios calculated via Poisson regression. To compare proportions, we used the chi-square or mean score test, while medians were compared by the Mann-Whitney test. SAS version 9.2 (SAS Institute) was used for all analyses.

To estimate the total effect of HAART on mortality, one must adjust for confounders measured at baseline as well as time-varying confounders affected by prior exposure, factors that are causal intermediates between treatment and death while simultaneously common causes of subsequent treatment and death [31]. We did so by fitting four logistic regression models to predict subject- and time-specific probabilities of treatment and censoring as a function of covariate histories, and using these predicted probabilities to construct stabilized inverse probability of treatment and censoring (IPTC) weights. The model for the denominator of the treatment weight included baseline confounders, time-varying confounders affected by exposure, and time as independent variables, while the model for the treatment weight numerator included only time and baseline confounders. The models for the censoring weight numerator and denominator were identical to those for the treatment weights, except that the censoring models also included time-varying HAART as a predictor. Then, these IPTC weights were used in a weighted pooled logistic model, which included baseline confounders, to estimate the parameters of a Cox proportional hazards marginal structural model [32],[33]. Assuming no unmeasured confounding, informative censoring, or model misspecification, weighting disassociates time-varying confounders with subsequent treatment and censoring, effectively eliminating the causal intermediates complicating estimation of the effect of HAART on mortality. With the intent-to-treat assumption that children starting HAART received it uninterruptedly throughout follow-up—approximately true in our program because adherence was checked at every visit and treatment was never discontinued for active patients—the marginal structural model yields a hazard ratio (HR) comparing the hazard of death had all children initiated HAART to the hazard of death had no children initiated HAART during follow-up.

For comparison, we also fit unadjusted as well as adjusted but unweighted pooled logistic models. Additionally, to address possible bias resulting from the dynamic visit schedule [34], we utilized an alternative weighted model that employed the above described IPTC weight multiplied by a visit attendance weight, that is, an inverse probability of treatment, censoring, and visit attendance (IPTCV) weight. The models for the probability of having a visit were equivalent to the censoring weight models, except that time since last visit was also included as a predictor. The constancy of HRs over follow-up was assessed with models that included an interaction term between HAART and categorical time. Kaplan-Meier curves to visualize the impact of HAART on survival were constructed; unstabilized weights were used to obtain unconditional, unstratified IPTCV-weighted curves [35]. The complements of Kaplan-Meier curves were plotted as estimates of cumulative incidence. 95% confidence intervals (CIs) for marginal structural model HRs were based on robust variance to account for within-subject correlation induced by weighting. Our pooled dataset included one row per person-day, with missing covariate data carried forward from the last observation.

Confounders were selected based on a posited causal directed acyclic graph [36] and previous studies. Baseline confounders were WHO HIV clinical stage and severity of immunodeficiency, age, and gender; time-varying confounders affected by prior exposure were cotrimoxazole prophylaxis, HIV-related symptoms or conditions, and CD4 cell percentage. HAART, gender, cotrimoxazole, and symptoms or conditions were coded dichotomously. HIV-related symptoms or conditions included one or more of the following: Kaposi's sarcoma, oral or esophageal candidiasis, severe weight loss, tuberculosis, fever or diarrhea lasting 1 mo or more, lymphocytic interstitial or *Pneumocystis jirovecii* pneumonia, chronic herpes simplex, oral hairy leukoplakia, cryptococcal meningitis, toxoplasma or HIV encephalopathy, or HIV-associated nephropathy. Because it was generally assessed only at enrollment, clinical stage was that at HIV care initiation for all children, including those for whom follow-up began at first CD4 percentage result. Severity of immunodeficiency was calculated according to WHO guidelines [27] using CD4 and age at baseline. Both clinical stage and severity of immunodeficiency were coded into four levels and treated as indicator variables in multivariable analyses. CD4 percentage, age, and time were modeled as restricted cubic splines with four knots, at the 5^th^, 35^th^, 65^th^, and 95^th^ percentiles.

## Results

Characteristics of the 790 children at baseline are shown in Table 1. The median age was 5.9 y (interquartile range [IQR] 2.7–9.8), and roughly one-half were female (52.5%). The majority of patients had severe immunodeficiency (57.2%), as reflected in the low median CD4 percentage of 15 (IQR 9–22). Most children had advanced HIV, as indicated by clinical stage 3 or 4 (51.3%), and 19.9% had evidence of at least one HIV-related symptom or condition.

**Table 1:** Characteristics of 790 HIV-infected children initiating HIV care in Kinshasa, DRC, between December 2004 and May 2010.

Time Period	Characteristic	Subcategory	Total (*n* = 790)	Initiated HAART (*n* = 619)	No HAART (*n* = 171)	*p*-Value^a^
**Baseline** b	Median age, years (IQR)		5.9 (2.7–9.8)	5.9 (2.6–9.6)	5.9 (3.4–10.2)	0.17
	Age, *n* (%)	<1	63 (8.0)	52 (8.4)	11 (6.4)	0.76
		1–4	277 (35.1)	218 (35.2)	59 (34.5)	
		5–9	265 (33.5)	208 (33.6)	57 (33.3)	
		10–17	185 (23.4)	141 (22.8)	44 (25.7)	
	Female sex, *n* (%)		415 (52.5)	314 (50.7)	101 (59.1)	0.05
	HIV clinical stage (WHO), *n* (%)	1	153 (19.4)	80 (12.9)	73 (42.7)	<0.01
		2	232 (29.4)	176 (28.4)	56 (32.7)	
		3	369 (46.7)	331 (53.5)	38 (22.2)	
		4	36 (4.6)	32 (5.2)	4 (2.3)	
	Median CD4 percentage (IQR)		15 (9–22)	13 (7–20)	22 (16–28)	<0.01
	Severity of immunodeficiency (WHO), *n* (%)	Not significant	174 (22.0)	100 (16.2)	74 (43.3)	<0.01
		Mild	88 (11.1)	56 (9.0)	32 (18.7)	
		Advanced	76 (9.6)	57 (9.2)	19 (11.1)	
		Severe	452 (57.2)	406 (65.6)	46 (26.9)	
	HIV symptoms or conditions, *n* (%)		157 (19.9)	139 (22.5)	18 (10.5)	<0.01
	Started cotrimoxazole at first visit, *n* (%)		726 (91.9)	575 (92.9)	151 (88.3)	0.05
**Follow-up**	Total person-years accrued		2,089.8	1,832.8	257.0	N/A
	HAART person-years accrued		1,620.9	1,620.9	0.0	N/A
	Median months of follow-up (IQR)		31.2 (10.3–53.6)	36.9 (14.0–55.7)	11.5 (3.0–27.0)	<0.01
	Median number of program visits (IQR)		30 (11–57)	40 (16–61)	9 (4–18)	<0.01
	Lost to follow-up or transferred care, *n* (%)		82 (10.4)	49 (7.9)	33 (19.3)	<0.01
	Died, *n* (%)		80 (10.1)	51 (8.2)	29 (17.0)	<0.01

a *p*-Values are for the comparison of children who received HAART to children who did not receive HAART.

b Baseline was date of first CD4 percentage result for 41 of 790 children (5.2%) for whom CD4 percentage at enrollment was not available. For these 41 children, the median number of months from enrollment to first CD4 percentage was 2.3 (IQR 1.1–5.3).

The 790 children, 619 of whom initiated HAART (78.4%) during follow-up, were followed for a median of 31.2 mo (IQR 10.3–53.6) and had a median of 30 HIV care visits (IQR 11–57). Of those who started treatment, 110 (17.8%) switched to an alternative regimen because of an adverse event or treatment failure. At baseline, compared to those who remained untreated, children who initiated HAART later had a greater degree of immunodeficiency (*p<*0.01) with a corresponding lower median CD4 percentage (*p<*0.01), had a more advanced HIV clinical stage (*p<*0.01), and were more likely to have at least one HIV-related symptom or condition (*p<*0.01). Those who initiated HAART were similar to those who did not in terms of gender (*p = *0.05) and median age (*p = *0.17), as well as cotrimoxazole initiation at the beginning of follow-up (*p = *0.05). The median duration of observation for children who started HAART, 36.9 mo (IQR 14.0–55.7), with 31.3 of those months (IQR 11.4–52.0) during receipt of HAART, was longer than the median 11.5 mo (IQR 3.0–27.0) observed for untreated children (*p<*0.01), and there was a parallel difference in median number of visits (40 versus 9, *p<*0.01).

**Figure 1:**
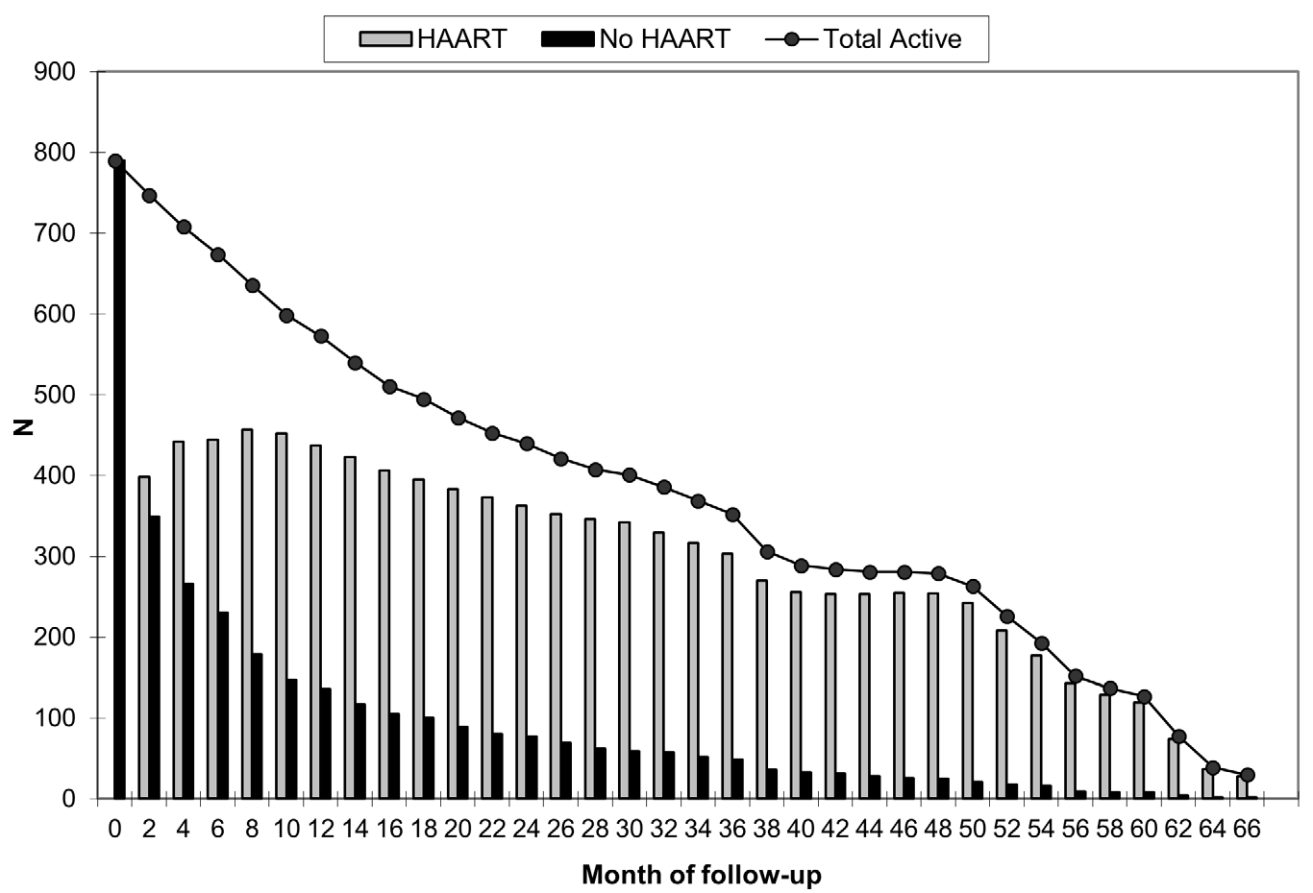
Number of active children by month of follow-up and HAART status. The timing of HAART initiation, in addition to the reduction in population size over time due to death, transfer to a different care provider, or loss to follow-up, is illustrated.

Eighty children (10.1%) died during the 2,089.8 accrued person-years of follow-up, an overall mortality rate of 3.8 deaths per 100 person-years (95% CI 3.1–4.8). The unadjusted mortality rate ratio comparing HAART to no HAART was 0.54 (95% CI 0.34–0.85). There were 51 deaths during the 1,620.9 person-years (77.6% of total follow-up) contributed by children receiving HAART, a rate of 3.2 deaths per 100 person-years (95% CI 2.4–4.2), and 29 deaths during the 468.9 non-HAART person-years, a rate of 6.0 deaths per 100 person-years (95% CI 4.1–8.6). The mortality rates per 100 person-years during and after the first 90 d of HAART were 16.4 (95% CI 11.0–24.5) and 1.8 (95% CI 1.3–2.7), respectively. The proportion of HAART-untreated children who died (17.0%) was higher than the 8.2% of HAART-initiating children who died (*p<*0.01), and the absolute 3-y risk of death for children receiving HAART was 0.14 compared with 0.48 for children not receiving treatment. Thus, the unadjusted HAART mortality risk ratio was 0.31 (95% CI 0.23–0.43). Six children (0.8%) transferred to a different care provider, and 76 (9.6%) were lost to follow-up. A smaller proportion of children who initiated HAART (7.9%) than untreated children (19.3%) either transferred to a different care provider or were lost to follow-up (*p<*0.01), suggesting that HAART, with its associated more frequent visit schedule, improved retention in care, and reflecting that children who were in care for a shorter period of time had less opportunity to begin treatment. Figure 1, in addition to showing the reduction in population size over time due to death, transfer of care, or loss to follow-up, illustrates the timing of HAART initiation. Of the 619 children who initiated treatment, 325 (52.5%) did so during the first 30 days of follow-up.

**Figure 2:**
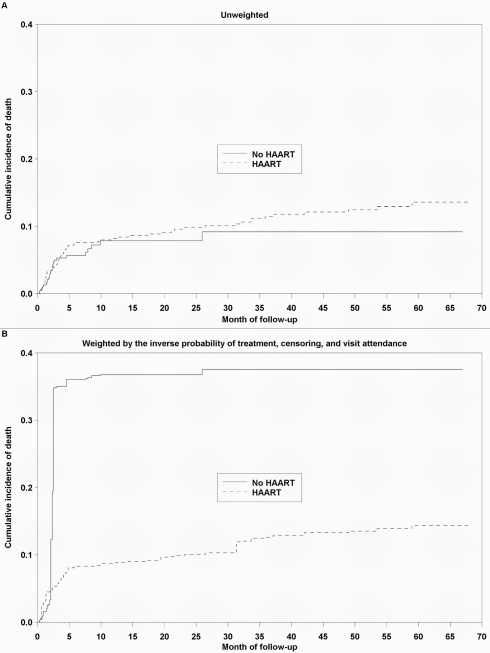
Cumulative incidence curves depicting the effect of HAART on survival among 790 HIV-infected children. In (A), the curves are unweighted. In (B), the curves are weighted by the IPTCV.

Estimates of the effect of HAART on mortality are presented in Table 2. Because patients with advanced disease are most likely to begin treatment, an unadjusted model that did not account for this confounding by indication suggested no effect of HAART on mortality, relative to no HAART (HR 1.38, 95% CI 0.84–2.27). An unweighted model that adjusted for baseline confounders only (HR 0.73, 95% CI 0.41–1.31), as well as an unweighted model that additionally but improperly adjusted for time-varying confounders (HR 0.67, 95% CI 0.37–1.21), shifted the estimates in the direction of the null but also failed to clearly suggest a protective effect of HAART on survival. After appropriately accounting for time-dependent confounders affected by exposure using marginal structural models, HAART was strongly protective against mortality. The HR from an IPTC-weighted model was 0.17 (95% CI 0.05–0.64), while that from an IPTCV-weighted model was 0.25 (95% CI 0.06–0.95). The HR from an IPTCV-weighted model before the median event time of 2.8 mo, 0.29 (95% CI 0.09–0.95), was similar to the HR of 0.18 (95% CI 0.03–0.99) after the median (interaction *p = *0.32). Advanced and severe immunodeficiency at baseline, compared to not significant immunodeficiency, were the only baseline factors independently associated with mortality in an IPTCV-weighted model (HR 3.16, 95% CI 1.18–8.47, and HR 5.08, 95% CI 1.49–17.39, respectively). IPTCV-weighted cumulative incidence curves depict the survival benefit of HAART, not evident in the unweighted curves that do not adjust for confounding and selection bias (Figure 2).

**Table 2:** Estimated effect of HAART on mortality among 790 HIV-infected children initiating HIV care in Kinshasa, DRC, between December 2004 and May 2010.

Model	HR	Robust 95% CI
Unweighted, unadjusted (no confounders)	1.38	0.84–2.27
Unweighted, adjusted (baseline confounders only)	0.73	0.41–1.31
Unweighted, adjusted (baseline and time-varying confounders)	0.67	0.37–1.21
IPTC-weighted	0.17	0.05–0.64
IPTCV-weighted	0.25	0.06–0.95

All estimates are derived from pooled logistic models that include time modeled as a restricted cubic spline with four knots. Comparing HAART to no HAART, the unadjusted mortality rate ratio was 0.54 (95% CI 0.34–0.85), while the unadjusted ratio of 3-y mortality risks was 0.31 (95% CI 0.23–0.43).

## Discussion

This study, which uniquely uses observational data to estimate the effect of HAART on mortality in a pediatric population in a resource-deprived setting, revealed that treatment markedly improved the survival of HIV-infected children in Kinshasa, DRC. Using a marginal structural model, we estimated that HAART reduced the hazard (rate) of mortality during follow-up, relative to no HAART, by 75% (HR 0.25, 95% CI 0.06–0.95). Although it was less precise, our result was essentially identical in magnitude to the HR of 0.24 (95% CI 0.11–0.51) from the only other study in children [12] to use a method that could overcome barriers to unbiased effect estimation in non-randomized data, such as time-dependent confounders affected by exposure. This is particularly noteworthy since the earlier study included children enrolled in a multicenter prospective cohort study in the United States. Combining the results from the two studies in a mini meta-analysis by inverse variance weighting, the HR was 0.24 with a 95% CI of 0.12–0.47.

The equivalence of these HRs offers evidence that HAART is as effective in improving the survival of HIV-infected children in the DRC, a severely resource-deprived nation still recovering from its recent history of poor governance and civil wars [37], as it is in resource-privileged settings. This result parallels the consistency of effect observed between adults in Europe and the United States [38] and South Africa [39], and is important because virtually all children receiving HAART around the world, because of scale-up of antiretroviral provision in low- and middle-income countries, live in resource-poor areas [13]. Pediatric HIV programs in sub-Saharan Africa, like ours in Kinshasa, often face challenges that could adversely affect patient survival, including limited drug options and diagnostic capacity, delayed healthcare seeking and poor retention in care, and prevalent comorbidities and undernutrition [40]. These factors emphasize why a homogeneous effect of HAART on survival in children could not be assumed across different settings. Evidence of the interaction between HAART and nutrition is limited, but it has been speculated that micronutrient deficiencies may decrease the effectiveness of antiretroviral drugs [41]. If true, this might have resulted in effect estimates that were attenuated compared to the case where children were better nourished overall, although it makes the results applicable to similar populations. The higher mortality rate observed during the first 90 d of HAART, compared to the rate after 90 d of therapy, was consistent with findings in multiple prior pediatric studies [21]–[25].

The validity of results is contingent upon methodological assumptions, one of which is the inclusion of all the important confounders. Although HIV viral loads were infrequently assessed, it is arguable that this factor, when available, was prognostic for and affected by HAART, as well as associated with survival. While not including viral load is a limitation that may have introduced some bias into our estimates, failing to account for this factor may have had only minimal effect. In the study by Patel et al. [12], the HR of 0.30 (95% CI 0.11–0.82) in the 36% of children with HIV RNA measurements was similar to the overall HR, which was not adjusted for viral load: 0.24 (95% CI 0.11–0.51). Limited availability of second- and third-line drug options, and being able to measure virological suppression only occasionally, also meant that some children who were recorded as receiving treatment could have actually been failing therapy (i.e., their treatment was not effective). This may have diluted the estimated effect of HAART if these patients were more likely to die. However, because children were frequently evaluated both clinically and immunologically, and because adherence was routinely assessed, it is expected that inadequate virological control was uncommon.

As suggested by several recent pediatric studies [21],[24],[42], it is conceivable that some children classified as lost to follow-up had died, which might have affected both the validity and precision of our estimate. If this was more common amongst children not receiving HAART, possibly because there was less opportunity to discern deaths in these children owing to their less frequent clinic visits, the strength of effect could have been underestimated. Because children not receiving HAART had fewer appointments, their data were correspondingly carried forward to a greater degree, which also may have affected results. On a related note, covariate data were quite complete. The variable most often missing, because it required laboratory testing and not just simple collection onto forms during clinic visits, was CD4 cell percentage. Assuming that children were on average halfway to their next scheduled test when follow-up concluded, 83% of the expected number of results were available. Data on other time-varying factors were close to fully complete. It is important to emphasize that the methods used will not yield a fully unbiased measure unless all underlying assumptions are met. If study imperfections occurred (e.g., misclassification of HAART status, or the incorrect recording of HIV-related symptoms or imprecise diagnosis of certain conditions such as cryptococcal meningitis, which may have occurred due to the limited capacity in Kinshasa), this is a possibility.

Strengths of our study included precise ascertainment of dates of HAART initiation and death, regular patient follow-up, which mitigated the influence of the carrying forward of data, and no treatment discontinuation by any active patient, which allowed for the intent-to-treat assumption and straightforward effect interpretation. In our setting, patients who missed visits were actively tracked, which decreased the possibility that children who died were misclassified as lost to follow-up. This is supported by the fact that during the first 90 d of HAART, a period when the rate of mortality was high (16.4 per 100 person-years, 95% CI 11.0–24.5), the rate of loss to follow-up was comparatively much lower (3.4 per 100 person-years, 95% CI 1.4–8.2). No child had ever received therapy prior to participation in our program, which adds to the generalizability and relevance of our results because most children initiating HAART worldwide are treatment-naïve. Furthermore, given the brief history and limited availability of vertical transmission prevention services in Kinshasa, it is unlikely that many children had been previously exposed to prophylactic NVP. This means that NVP-resistant HIV variants [43],[44] were probably rare, and increases the likelihood that the NVP-based regimens most children received were effective. Patel et al. [12] sensibly speculate that a stronger HR than 0.24 would have been estimated had their population been naïve to treatment rather than experienced. If true, because our HR of 0.25 was comparable despite arising from treatment-naïve children, it is possible that the aforementioned underlying contextual factors may have moved our estimate modestly towards the null.

The large difference between the HRs from the unweighted and weighted models indicates that there was considerable time-dependent confounding, and validates the analytical approach. The importance of accounting for dynamic visit schedules is highlighted by the distinct HRs from the IPTC- and IPTCV-weighted models.

In summary, HAART had a substantial beneficial impact on the survival of HIV-infected children in Kinshasa, DRC. Along with supplementing the limited knowledge base on the quantitative effects of pediatric treatment, this study presents the first valid estimate, to our knowledge, of the extent to which therapy decreases mortality in a highly relevant population of HIV-infected children living in a resource-deprived setting. Our estimate, which represents the expected outcome had HAART been evaluated via randomized assignment, provides compelling evidence that the accelerating rollout of antiretrovirals could save substantial numbers of lives. Pooled data from pediatric cohorts in less developed areas should be used in future research not only to generate more precise effect estimates, but also to focus on the effects of HAART within particular groups such as undernourished children, which remain incompletely understood.

## Abbreviations

CI: confidence interval
DRC: Democratic Republic of the Congo
HAART: highly active antiretroviral therapy
HR: hazard ratio
IPTC: inverse probability of treatment and censoring
IPTCV: inverse probability of treatment, censoring, and visit attendance
IQR: interquartile range
NVP: nevirapine
WHO: World Health Organization
